# The Organization of the Sinoatrial Node Microvasculature Varies Regionally to Match Local Myocyte Excitability

**DOI:** 10.1093/function/zqab031

**Published:** 2021-06-12

**Authors:** Nathan Grainger, Laura Guarina, Robert H Cudmore, L Fernando Santana

**Affiliations:** Department of Physiology and Membrane Biology, University of California, Davis, Davis, CA 95618, USA; Department of Physiology and Membrane Biology, University of California, Davis, Davis, CA 95618, USA; Department of Physiology and Membrane Biology, University of California, Davis, Davis, CA 95618, USA; Department of Physiology and Membrane Biology, University of California, Davis, Davis, CA 95618, USA

**Keywords:** vasculature, HCN, calcium, sarcoplasmic reticulum, stochastic resonance, pacemaking

## Abstract

The cardiac cycle starts when an action potential is produced by pacemaking cells in the sinoatrial node. This cycle is repeated approximately 100 000 times in humans and 1 million times in mice per day, imposing a monumental metabolic demand on the heart, requiring efficient blood supply via the coronary vasculature to maintain cardiac function. Although the ventricular coronary circulation has been extensively studied, the relationship between vascularization and cellular pacemaking modalities in the sinoatrial node is poorly understood. Here, we tested the hypothesis that the organization of the sinoatrial node microvasculature varies regionally, reflecting local myocyte firing properties. We show that vessel densities are higher in the superior versus inferior sinoatrial node. Accordingly, sinoatrial node myocytes are closer to vessels in the superior versus inferior regions. Superior and inferior sinoatrial node myocytes produce stochastic subthreshold voltage fluctuations and action potentials. However, the intrinsic action potential firing rate of sinoatrial node myocytes is higher in the superior versus inferior node. Our data support a model in which the microvascular densities vary regionally within the sinoatrial node to match the electrical and Ca^2+^ dynamics of nearby myocytes, effectively determining the dominant pacemaking site within the node. In this model, the high vascular density in the superior sinoatrial node places myocytes with metabolically demanding, high-frequency action potentials near vessels. The lower vascularization and electrical activity of inferior sinoatrial node myocytes could limit these cells to function to support sinoatrial node periodicity with sporadic voltage fluctuations via a stochastic resonance mechanism.

## Graphical Abstract

**Graphical Abstract:**
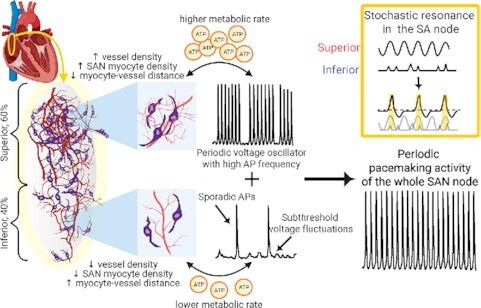

## Introduction

The function of the sinoatrial node (SAN) is to produce the action potentials (APs) that initiate each heartbeat. These APs are generated by clusters of pacemaker cells firing in unison^1^ and propagate via gap junctions to neighboring SAN myocytes and, eventually, surrounding atrial myocytes.^2^ SAN myocytes that are firing APs at the highest frequency become the dominant leading pacemaker site.

The mechanisms underlying pacemaking activity in SAN myocytes have been the subject of intense investigation.^3–9^ Initiation of the AP occurs during diastole and is driven by activation of hyperpolarization-activated cyclic nucleotide-gated currents.^6, 10–13^ HCN4 is the predominant HCN channel in the node, but HCN1-2 are also expressed. ^10, 11^ Concurrently, spontaneous, stochastic local sarcoplasmic reticulum (SR) Ca^2+^ release events via ryanodine receptors (ie, Ca^2+^ sparks)^14^ activate inward diastolic Na^+^/Ca^2+^ exchanger currents.^4,15^ These HCN and Na^+^/Ca^2+^ exchanger currents depolarize SAN myocytes over the threshold of activation of voltage-gated Ca_V_3.1,^16^ Ca_V_1.3,^17,18^ and, eventually, Ca_V_1.2 channels,^18,19^ thereby initiating the AP and triggering a transient global increase in intracellular Ca^2+^ concentration ([Ca^2+^]_i_). Membrane hyperpolarization begins by the inactivation of Ca^2+^ currents and activation of voltage-gated K^+^ currents. As SAN myocytes approach the maximum diastolic potential, HCN channels are activated, initiating the whole process again. APs propagate within the SAN via gap junctions,^20^ eventually reaching surrounding atrial myocytes.

The dominant pacemaking site in the SAN is not static.^19,21,22^ Instead, it dynamically shifts within the SAN in response to physiological stimuli, including activation of the autonomic nervous system.^2^ A recent study on ex vivo rat and human SAN tissue supports the concept that 2 distinct pacemaker sites coinhabit the SAN, one near the superior section of the node and another further below in the inferior section.^23^ Depending on the degree of sympathetic and parasympathetic nerve activity, the leading pacemaker site in the SAN shifts between superior and inferior regions. With high sympathetic drive, the superior SAN functions as the dominant pacemaking center. The pacemaker center shifts toward the inferior SAN during strong parasympathetic activation. Accordingly, a shift in pacemaker origin has been proposed to trigger changes in heart rate.^23^

Irrespective of pacemaker location, SAN myocytes require energy in the form of adenosine triphosphate (ATP) for normal function. For example, significant amounts of ATP are consumed for the maintenance of Na^+^ and K^+^ gradients across the sarcolemma by the Na^+^/K^+^ pump, critical for Na^+^/Ca^2+^ exchanger function. The SR Ca^2+^ pump, responsible for refilling the internal Ca^2+^ store during each beat, also requires a significant amount of energy to operate in muscle.^24^ Although SAN myocytes do not have as many sarcomeres as atrial and ventricular myocytes, they still consume ATP during crossbridge cycling. Furthermore, the constant generation of cytosolic adenosine monophosphate (cAMP), fulfilled by the plasma membrane-associated enzyme adenylate cyclase, is an energetically demanding process that consumes significant amounts of ATP.^25^ cAMP is required for protein kinase A–dependent phosphorylation of multiple proteins, including HCN and Ca^2+^ channels as well as SR Ca^2+^ pump.^12,26–29^ Even under basal conditions (ie, no autonomic stimulation), SAN myocytes consume more O_2_ compared with a paced (3 Hz) ventricular myocyte,^25^ demonstrating the relatively high metabolic demands of SAN myocytes. ATP consumption is elevated during increased sympathetic input to the SAN, when cAMP levels are further amplified, and AP frequency increased.^13^

To sustain the energetic requirements of SAN myocytes, the SAN artery, a branch of the right coronary artery, delivers oxygenated blood to the node. During diastole, when the compressive forces of the myocardium are at their lowest, blood moves through SAN artery and capillary network to deliver oxygen to pacemaker cells.^30^ Deoxygenated blood exits the SAN tissue via venules and specialized Thebesian veins where blood directly enters the right atrial lumen.^31^ In humans, the SAN artery has multiple anatomical variations^32^ and damage to this artery can be proarrhythmogenic.^33^ Despite the high metabolic demand of the SAN, a detailed understanding of the vascular network that supports the node for aerobic respiration is limited.

In this study, we used a combination of imaging approaches to generate high-resolution three-dimensional (3D) maps of SAN vascular anatomy and its relationship to SAN myocytes across the entire node. Analysis of these images shows that vascular density is high and myocyte-to-vessel distances are relatively short in the superior region of the node. However, vascular density decreases, sharply increasing myocyte-to-vessel distances toward the inferior region of the node. Using whole-cell patch-clamp techniques and Ca^2+^ imaging of isolated SAN myocytes from densely (ie, superior) and sparsely (ie, inferior) vascularized sections of the SAN, we discovered that superior-derived SAN myocytes are intrinsically capable of firing higher frequency APs versus inferior-derived SAN myocytes. Based on these data, we posit that blood supply could be a determinant in sculpting the firing properties of SAN myocytes, effectively determining the dominant pacemaking site within the SAN.

## Materials and Methods

### Animals

Male wild-type C57BL/6J mice (The Jackson Laboratory) aged between 6 and 14 weeks were used in this study. Mice were euthanized with a single, intraperitoneally administered lethal dose of sodium pentobarbital (250 mg/kg). All mice were maintained, and experiments conducted, in accordance with the University of California, Davis Institutional Animal Care and Use Committee guidelines.

### Whole-mount Immunohistochemistry

After confirmation of deep anesthesia, beating hearts were immediately removed and transferred to warmed (37°C) Tyrode III solution containing (in mm): 140 NaCl, 5.4 KCl, 1.0 MgCl_2_, 1.8 CaCl_2_, 5.0 HEPES, 5.5 d-glucose (pH = 7.4 with NaOH). The SAN region was dissected out of the heart and pinned flat. The SAN was briefly washed in phosphate-buffered saline then fixed in 4% paraformaldehyde in phosphate-buffered saline (0.01 M) for 30 min with gentle agitation. After fixation, the tissue was washed in phosphate-buffered saline (3 × 5 min) and then transferred to a 15 mL tube filled with phosphate-buffered saline and washed (12 h) on a low-speed tube rotator. To prepare fixed SAN tissues for labeling, tissues were dehydrated, cleared using ethanol in dimethyl sulfoxide and bleached to reduce autofluorescence as described previously.^34,35^ Briefly, SAN tissue was dehydrated through a graded ethanol series (25, 50, 75, 95, 100%), cleared for 2 h with dimethyl sulfoxide (20%) in ethanol and bleached for 12 h with hydrogen peroxide (6%) in ethanol. Tissue was then rehydrated through a graded ethanol series, washed in phosphate-buffered saline (3 × 5 min) and permeabilized with 0.5% Triton X-100 in phosphate-buffered saline (3 × 10 min). Nonspecific antibody binding was blocked by incubating SAN tissue for 2 h with 5% normal donkey serum in Triton X-100 phosphate-buffered saline. SAN tissue was washed in phosphate-buffered saline (3 × 10 min) and incubated for 48 h in a mix of primary antibodies in phosphate-buffered saline at 4°C (goat antimouse cluster of differentiation 31 protein [CD31], 1:50, AF3628 R&D Systems; rabbit antimouse HCN4, 1:200, AB5808 MilliporeSigma). After primary labeling, tissues were washed in phosphate-buffered saline (3 × 10 min) and labeled with secondary antibodies for 4 h in the dark at room temperature (donkey antirabbit Alexa Fluor 488, 1:1000, R37118, ThermoFisher Scientific; donkey antigoat Alexa Fluor 568, 1:1000, A11057, ThermoFisher Scientific). SAN tissue was then washed in phosphate-buffered saline (3 × 10 min) and incubated for 2 h in a dimethyl sulfoxide and phosphate-buffered saline mixture (1:4). Tissues were then mounted in Aqua-Mount mounting medium (Thermo Scientific) and cover slipped.

### Immunohistochemistry Image Acquisition and Analysis

Imaging of fixed SAN tissues was performed using an inverted confocal laser scanning microscope (Olympus FluoView FV3000, Olympus Corporation, Tokyo, Japan) with a 40× oil-immersion lens (Olympus APON 40×, numerical aperture = 1.3) and acquired using commercial software (Olympus FluoView). Images were acquired at 0.5 µm/pixel with a field of view of 320 µm. Image volumes consisted of between 80 and 120 *z*-planes. In order to image the entirety of each SAN preparation, a mosaic of 3D image volumes were acquired. All image analysis was performed using a combination of Imaris 9.7 (Oxford Instruments, Abingdon, U.K.), ImageJ/Fiji^36^, and Python (3.7). Both vascular (CD31-positive, CD31^+^) and myocyte (HCN4-positive, HCN4^+^) image volume color channels were thresholded to generate 3D binary masks. When necessary, CD31^+^ positive endocardium was manually removed from the image volumes using Imaris. Vascular and myocyte fractional volumes were calculated from 3D binary masks with custom-written Python scripts, with fractional volume = (number of pixels in the 3D mask)/(total number of pixels in the volume). The 3D binary masks were used to calculate the 3D distance from each HCN4^+^ pixel to the nearest CD31^+^ pixel (a Euclidean distance transform, using the SciPy Python package). Three-dimensional masking of HCN4^+^ and CD31^+^ volumes was repeated using either Imaris or Python and obtained similar results. For 3D masking in Python, The Allen Institute for Cell Science—Segmentation package (https://github.com/AllenInstitute/aics-segmentation) was used. For 3D vascular segmentation, the total vessel segments and length per field of view were calculated using Imaris. The diameters of 1°–4° vessels were manually traced in ImageJ/Fiji using CD31^+^ image volumes.

### Single SAN Myocyte Isolation

Following euthanasia, SAN tissue was immediately dissected from the beating heart and placed in warmed Tyrode III solution, containing (in mm) 140 NaCl, 5.4 KCl, 1 MgCl_2_, 5 HEPES, 1.8 CaCl_2_, 5.5 glucose (pH 7.4 with NaOH). The SAN was pinned flat and superior and inferior regions identified. The SAN myocyte isolation described in this protocol is based on a modified version of the SAN isolation detailed by Fenske et al.^37^ Briefly, intact tissue pieces derived from the superior and inferior SAN regions (approx. 2–3 mm^2^ each) were bathed for 5 min at 36°C in Tyrode low Ca^2+^ solution containing (in mm): 140 NaCl, 5.4 KCl, 0.5 MgCl_2_, 0.2 CaCl_2_, 5.0 HEPES, 5.5 d-glucose, 1.2 KH_2_PO_4_, 50 Taurine (pH = 6.9 with NaOH). Tissue regions were then subjected to enzymatic digestion for 30 min at 36°C in Tyrode low Ca^2+^ solution (pH 6.9) containing: 9.43 U elastase, 0.89 U protease, 0.27 U collagenase B and bovine serum albumin (1 mg/mL). During enzymatic digestion, tissue segments were gently agitated every 5–7 min. Once the digestion was complete, tissue segments were rinsed twice with Tyrode low Ca^2+^ solution (pH 6.9) and twice with an ice-cold Kraft-Brühe solution (80 mml-glutamic acid, 25 mm KCl, 3 mm MgCl_2_, 10 mm KH_2_PO_4_, 20 mm Taurine, 10 mm HEPES, 0.5 mm ethylene glycol-bis(2-aminoethylether)-*N,N,N′,N′*-tetraacetic acid, 10 mm glucose, pH 7.4 with KOH) and allowed to rest at 4°C in approximately 500 μL of the Kraft-Brühe solution. After 2–3 h at 4°C, the tissue was then warmed to 37°C for 5–10 min and mechanically dissociated using a fire polished glass pipette.

### Perforated Patch Clamp Recordings

A drop of cell suspension was placed on a temperature-controlled recording chamber (35–36°C) and cells were allowed to settle and adhere to the chamber for approximately 10 min. Next, the external Ca^2+^ concentration was slowly risen by adding increasing amounts of Tyrode solution^37^ to allow a graded transition to physiological Ca^2+^ levels (ie, 1.8 mm). After completion of Ca^2+^ reintroduction, cells were constantly perfused with Tyrode solution. The internal solution used for current clamp recordings contained (in mm): 125 K-Aspartate, 10 NaCl, 15 KCl, 1 CaCl_2_, 10 HEPES (pH to 7.2 with KOH). Before commencement of patch clamp recordings, 100 μm amphotericin B was added to the internal pipette solution and the internal solution was kept on ice. Fire-polished glass pipettes were first dipped in an amphotericin B-free internal solution and then back filled with the amphotericin B–containing internal solution.

Electrophysiological signals were recorded using an amplifier (MultiClamp 700B, Molecular Devices, San Jose, CA) controlled with pCLAMP 10 software (Molecular Devices). Cells were initially patched in voltage-clamp and upon the formation of a giga-ohm seal, a short period of time was required (1–5 min) until amphotericin B permeabilized the membrane, allowing electrical access to the cell. Accessed cells were transitioned to current-clamp mode to record membrane voltage. All recordings were acquired at 10 kHz.

All current-clamp recordings were analyzed using custom Python scripts for the detection and analysis of SAN myocytes AP parameters as previously described.^38^ The take-off potential was defined as the membrane potential when the first derivative of voltage with respect to time (dV/dt) reached 10% of its maximum value. The take-off potential was used as the time of an AP and to calculate both AP frequency (Hz) and inter–AP interval (ms). The maximum depolarizing potential was defined as the most negative membrane potential between APs. The diastolic duration was defined as the interval between the maximum depolarizing potential and take off potential. The early diastolic depolarization rate was estimated as the slope of a linear fit between 10% and 50% of the diastolic duration and the early diastolic duration was the corresponding time interval.

To detect subthreshold membrane fluctuations, we used the “threshold search” function of Clampfit (Molecular Devices). A subthreshold voltage fluctuation was defined as an event that had an amplitude larger than the mean + 3 standard deviations of baseline voltage and a duration of at least 1 ms, but failed to exceed the experimentally determined AP take-off potential (ie, AP threshold) of –29 mV.

### [Ca^2+^]_i_ Imaging

After plating single SAN myocytes on the recording chamber, cells were loaded with the membrane-permeable acetoxymethyl-ester form of Fluo-4 (Fluo-4 AM, Invitrogen) for measurement of [Ca^2+^]_i_. Fluo-4 (5 µm) was mixed with Tyrode solution and introduced to plated cells during Ca^2+^ reintroduction (described above). Cells were loaded for 10 min, followed by a de-esterification period of 10 min. Temporal fluorescence fluctuations caused by [Ca^2+^] transients were acquired using the line-scan mode on an inverted confocal laser scanning microscope (Olympus FluoView 3000) with a 60× oil-immersion lens (Olympus APON 60×, numerical aperture = 1.49). Fluo-4 was excited with a 473 nm solid-state laser (Coherent Inc., Santa Clara, CA). Line-scan images were analyzed using ImageJ. Background subtracted fluorescence signals were normalized by dividing fluorescence at each point (F) with the baseline fluorescence (F_0_). Ca^2+^ sparks were identified automatically using the SparkMaster plugin of ImageJ.^39^

### Statistics

Parametric, normally distributed data are presented as mean ± standard error of the mean. The coefficient of variation was calculated by dividing the standard deviation by the mean of the dataset. In the case of nonparametric data, median and range values are reported. Group comparisons were made using a Student's *t*-test or analysis of variance (ANOVA) followed by a Tukey multicomparison test for parametric data. For nonparametric data, Mann–Whitney tests were implemented. A *P*-value of less than 0.05 was considered significant. The asterisk symbol is used in the figures to illustrate the significant difference between groups where NS (ie, not significant) represents *P* > 0.05; * represents *P* ≤ 0.05; ** represents *P* ≤ 0.01; and *** represents *P* ≤ 0.001.

## Results

### The Distribution of Vessels and SAN Myocyte Varies Along the SAN

**Figure 1.**
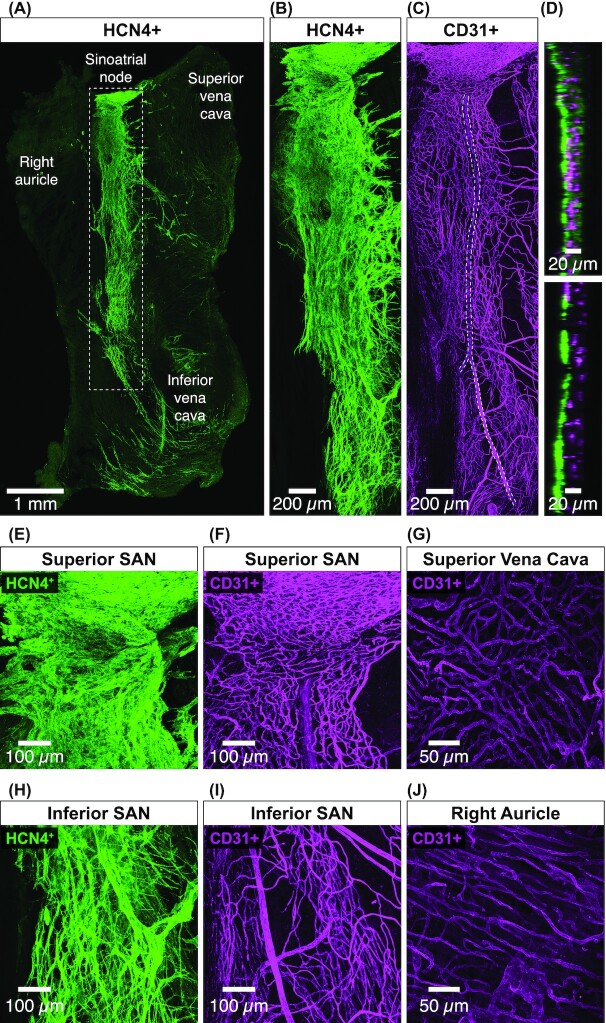
The distribution of vasculature and myocytes varies along the SAN. **(A)** An overview of the entire in vitro SAN preparation. Image is a maximal intensity projection of tiled 3D confocal images of HCN4^+^ SAN myocytes from a representative SAN (*n* = 6 SANs). The SAN runs from the superior vena cava to the inferior vena cava. The SAN is outlined (gray dotted rectangle). **(B)** Zoomed in maximal intensity projection of HCN4^+^ myocytes in the SAN region corresponding to the gray dotted rectangle in panel A. **(C)** Zoomed maximal intensity projection of CD31^+^ endothelial cells in the SAN region corresponding to the gray dotted rectangle in panel A. The length of the SAN artery is outlined (gray dotted line). **(D)** Orthogonal view of HCN4^+^ and CD31^+^ positive SAN myocytes and endothelial cells from the upper and lower regions of the SAN. Panels **(E)** and **(F)** show exemplar maximum intensity projection of the superior SAN HCN4^+^ myocytes CD31^+^ endothelial cells. **(G)** Representative maximum intensity projection of superior vena cava CD31^+^ endothelial cells. Panels **(H)** and **(I)** show representative maximal intensity projection of the inferior SAN HCN4^+^ myocytes and CD31^+^ endothelial cells. **(J)** Maximal intensity projection of exemplar right auricle CD31^+^ endothelial cells.

We investigated the distribution of vasculature and pacemaking myocytes along the SAN. To do this, we fixed and double labeled SAN tissue with an HCN4 antibody to reveal SAN myocytes and a CD31 (ie, cluster of differentiation 31 protein) antibody as a marker of endothelial cells (*n* = 6 SANs). We then used laser scanning confocal microscopy to image submicron resolution 3D stacks of HCN4-positive (HCN4^+^) SAN myocytes and CD31-positive (CD31^+^) endothelial cells within the entire SAN (Figure 1A).

Our initial inspection of these image volumes suggested regional variations in the density of both HCN4^+^ SAN myocytes (Figure 1B) as well as CD31^+^ vessels (Figure 1C). These regional heterogeneities are more evident in orthogonal images of SAN myocytes and endothelial cells from the superior and inferior sections of the SAN (Figure 1D). Note that in the superior SAN, vessels are embedded within SAN myocytes and become progressively segregated in the inferior SAN.

We zoomed into regions of the superior and inferior SAN to get a closer look at myocytes and vessels in these regions of the node. These images further suggest that myocyte and vessel density is highest in the superior SAN (Figure 1E/F), progressively decreasing in the inferior SAN (Figure 1H/I). These regional variations in myocyte distribution seems to be a unique feature of the SAN. Images of vessels in the superior vena cava (Figure 1G) and right auricle (Figure 1J) surrounding the SAN show that vessel density is homogeneous.

### The Distance Between SAN Myocytes and Vessels Varies Along the SAN

**Figure 2.**
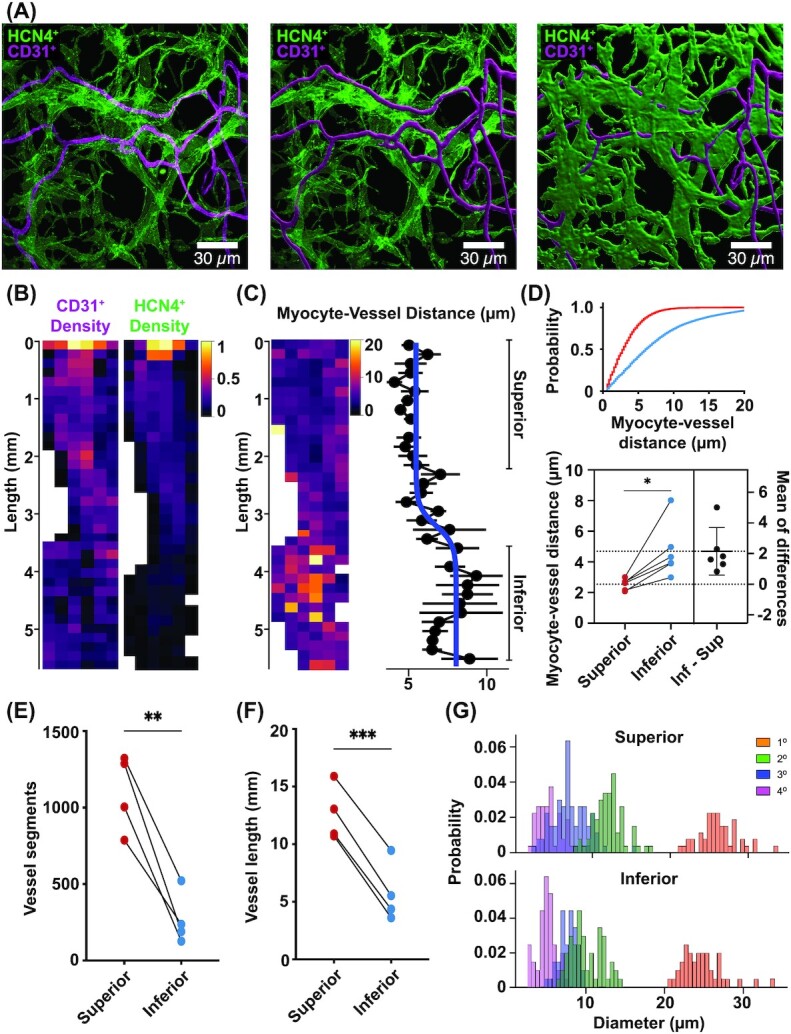
Vascular and myocyte densities are higher in the superior than the inferior SAN. **(A)** Exemplar maximum intensity projection image volumes showing the image analysis workflow. Starting with the raw image volumes of dual-labeled HCN4^+^ and CD31^+^ (left), the CD31^+^ channel is segmented to produce a vascular tracing (center). The HCN4^+^ channel is then segmented to produce a SAN myocytes mask (right). **(B)**Vessel (left) and myocyte (right) density heat maps across the entire image from an exemplar SAN preparation. Each pixel in the heat maps represents the (normalized) fractional volume occupied by the mask. **(C)** Myocyte-vessel distance heat map across the SAN preparation in panel B (left). Each pixel in the heat map represents the average distance from HCN4^+^ myocytes to CD31^+^ endothelial cells (µm). The plot (right) quantifies the mean ± standard error of the mean (µm) for each row in the heat map. A sigmoidal fit to quantitatively separate superior and inferior regions is overlaid (blue). **(D)** Plot shows the cumulative histogram distribution of myocyte-to-vessel distances (µm) in the superior (red) and inferior (blue) regions. Below, estimation plot of the average distances of myocytes-to-vessels in superior and inferior SAN (*n* = 6 SANs). **(E)**Plot of the total number of vessel segments in the superior and inferior SAN (*n* = 4 SANs, fields of view = 9 superior and 12 inferior regions). **(F)** Plot of the total vessel length (mm) in the superior and inferior SAN (*n* = 4 SANs, fields of view = 9 superior and 12 inferior regions), *P < 0.05. **(G)** Histogram of the diameters of 1°, 2°, 3°, and 4° order vascular branches in the superior and inferior regions of a representative SAN. *P ≤ 0.05, **P ≤ 0.01, ***P ≤ 0.001.

We performed a detailed analysis of HCN4^+^ myocytes and CD31^+^ vessels along the entire length of the SAN (Figure 2). To determine vascular and myocyte densities, we generated 3D masks of CD31^+^ endothelial cells and HCN4^+^ myocytes (see the "Materials and Methods" section). Masks of SAN myocytes and vessel masks were used to determine the distance of SAN myocytes to their nearest vessel. An example of this workflow is shown in Figure 2A.

To quantify the density of myocytes and vessels from the superior to inferior regions of the SAN, we calculated the fractional volume occupied by the CD31^+^ and HCN4^+^ masks and generated spatial heat maps encompassing the entire SAN (Figure 2B). We found the fractional volumes of both HCN4^+^ and CD31^+^ labeling decreases from superior to inferior regions.

What would the functional implications of this difference in vessel and myocyte density between superior and inferior SAN regions have for resident SAN myocytes? To address this, we calculated the 3D distance from each HCN4^+^ pixel to its nearest CD31^+^ positive pixel (a 3D Euclidean distance transform). This is an important parameter determining myocyte-vessel exchange and communication.^30,40,41^ Our analysis revealed increasing distances between myocytes and their neighboring vessels traversing from the superior to the inferior portions of the SAN (Figure 2C). In some regions of the inferior node, myocyte-vessel distance could exceed 10–15 μm.

We set out to develop a measure that would allow us to quantitatively segregate superior and inferior regions of the SAN. Fitting the mean myocyte-vessel distance with a sigmoidal function (Figure 2C, blue line) yields a separation of these 2 regions. We used this metric to separate superior and inferior regions for the remaining analysis.

To further reveal differences between the superior and inferior regions of the SAN, we performed additional analyses across a number of fixed-tissue SAN preparations and segregating each SAN imaging dataset into corresponding superior and inferior regions. A cumulative distribution histogram plotted for a single SAN preparation illustrates the difference in proximity of myocytes to vessels (top, Figure 2D). Indeed, we found that superior SAN myocytes were significantly closer to neighboring vessels (bottom, Figure 2D; superior 2.54 ± 0.4 µm; inferior 4.69 ± 1.8 µm, *P* = 0.016, *n* = 6 SANs).

Next, we quantified the number of vessel segments and total vessel length and compared the superior and inferior regions within each fixed SAN tissue. We averaged the number of vessel segments and total vessel length between different fields of view between each region and performed a pairwise analysis. This analysis reveals a significantly higher number of vessel segments in the superior versus inferior regions (Figure 2E; superior 1101 ± 126 segments; inferior 269 ± 87 segments, *P* = 0.0052, *n* = 4 SANs, fields of view = 9 superior and 12 inferior regions). Finally, we find that the superior region has a significantly higher total vessel length versus the inferior (Figure 2F; superior 12.6 ± 1.2 mm; inferior 5.7 ± 1.3 mm, *P* = 0.0001, *n* = 4 SANs, fields of view = 9 superior and 12 inferior regions).

Taken together, these results indicate a progressive decrease in both vessel and SAN myocyte densities along the primary axis of the SAN from the superior to the inferior venae cava. The functional effect is that SAN myocytes in the superior versus inferior region are in closer proximity to their neighboring vessels. This could impact the range of physiological signatures of SAN myocytes with superior cells receiving substantially more metabolic support via greater access to blood supply.

### Regional Variations in Vascular Density Along the SAN Are Not Due to Gross Vascular Topological Features

To determine if the observed differences in vascular density between the superior and inferior regions of the SAN were due to gross vascular topological features, we examined the branching patterns of the main SAN artery as it bifurcated into arterioles and then capillaries.

The main SAN artery arborizes in multiple locations as it descends the node giving rise to intermediate arterioles and finally capillaries (Figure 1C, F, and I). Interestingly, the diameter of the primary SAN artery (first order; 1°) was larger in the superior versus inferior regions (Figure 2G; superior 30.62 ± 4.3 µm, inferior 23.37 ± 3.9 µm, *P* = 0.02, *n* = 3 SANs). Given the decrease in caliber of the primary SAN artery from superior to inferior regions, we investigated if the secondary, tertiary, and quaternary vascular branches also varied between the superior and inferior regions. To do this, we segregated second-order (2°), third-order (3°), and fourth-order (4°) vessel segments in superior and inferior regions and calculated the diameter of each vessel segment. We saw no significant difference in the caliber of 2°, 3°, or 4° branches between the superior and inferior regions (2° *P* = 0.81, 3° *P* = 0.83, 4° *P* = 0.81, *n* = 3 SANs), indicating that although the primary SAN artery reduces in diameter between the superior and inferior regions, the downstream 2°, 3°, or 4° branches do not. Likewise, we did not observe any difference in the number of 2° or 3° order arteriole branches between the superior and the inferior SAN (superior 2 ± 1 branches, inferior 2 ± 1 branches, *P* = 1.0, *n* = 3 SANs).

We did observe a wide range of 2° and 3° branch diameters in both the superior (range for 2° was 7.6–16.7 µm and for 3° was 3.3–12.1 µm) and inferior (range for 2° was 8.1–16.6 µm and for 3° was 5.2–9.8 µm). This is in contrast to a narrow range of vessel diameters for 4° vessels (superior range was 3.1–7.6 µm, inferior range was 3.1–6.3 µm). From this analysis, we conclude that the 2° and 3° branches are likely arterioles, whereas the 4° branches are capillaries. Together, this analysis indicates that the gross vascular topological features does not differ between the superior and inferior SAN and in turn does not contribute to regional variations in vascular density.

### Regional Variations in AP Frequency SAN

Our data describing the SAN vasculature and its relationship to SAN myocytes have important functional implications. For example, the intrinsic electrical signaling modalities of SAN myocytes in the superior could be fundamentally similar but vary in vivo in response to differential blood supply. Alternatively, the firing properties of SAN myocytes themselves may vary along the SAN. One plausible hypothesis is that the superior SAN is populated by myocytes that have a higher intrinsic capacity to fire APs than the inferior SAN. In this model, regional variations in AP firing patterns in vivo would likely be due to differences in intrinsic excitability as well as blood supply.

As a first step to test these hypotheses, we developed protocols to identify, dissect, and enzymatically dissociate myocytes from the superior and inferior SAN. We used our vessel and myocyte density data (see Figures 1 and 2) to anatomically define the superior and inferior SAN. We chose a region of approximately 2–3 mm^2^ for the superior SAN and similar area for the inferior SAN and dissociated cells from each region individually.

**Figure 3.**
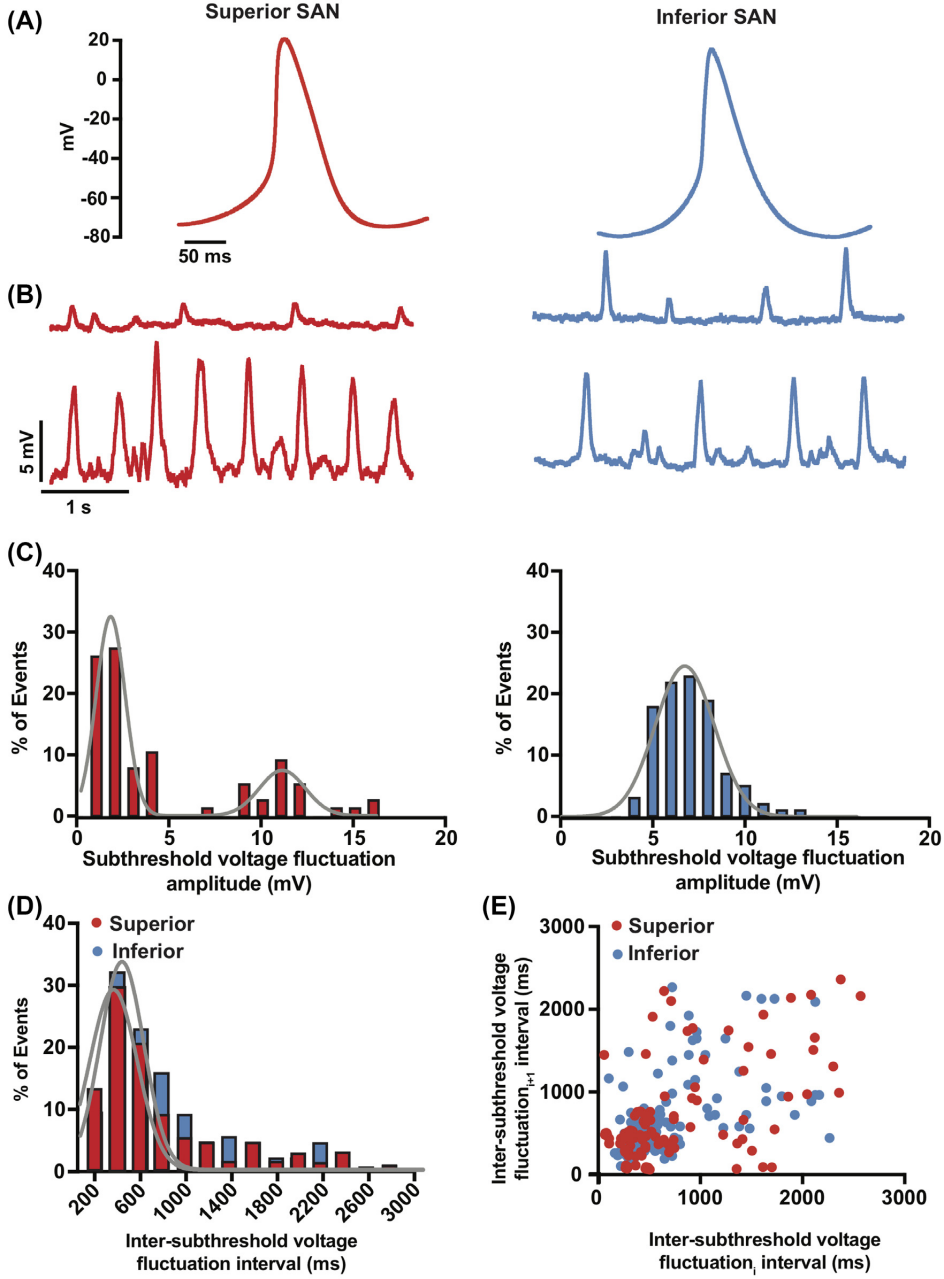
AP waveform and the amplitude of stochastic subthreshold voltage fluctuations vary in superior and inferior SAN myocytes. **(A)** Representative AP traces from superior (left) and inferior (right) SAN myocytes. **(B)** Record of subthreshold membrane fluctuations from exemplar superior (left) and inferior (right) SAN myocytes. **(C)** Histograms of subthreshold voltage fluctuations from superior (left, *n* = 93 subthreshold voltage fluctuations from 5 cells) and inferior (right, *n* = 81 subthreshold voltage fluctuations from 6 cells) SAN myocytes. The histogram from superior SAN myocytes was fit with the sum of 2 Gaussian functions (solid gray lines). The histogram from inferior SAN myocytes was best fit with a single Gaussian function (solid gray line). **(D)** Histograms of distribution of interevent intervals of subthreshold voltage fluctuations in superior (red bars) and inferior (blue bars) SAN myocytes. The histograms from superior and inferior SAN myocytes were each fit with a single Gaussian function (solid gray lines). **(E)** Scatter plot of the subthreshold voltage fluctuation interval (inter-subthreshold voltage fluctuations interval_i_) on the *x*-axis versus the interval of the subsequent subthreshold voltage fluctuation (inter-subthreshold voltage fluctuations interval_i+1_) in superior (red circles) and inferior (blue circles) SAN myocytes.

We performed whole-cell perforated-patch current-clamp recordings in individually isolated superior and inferior SAN myocytes (Figure 3). Figure 3A shows single AP records from representative superior and inferior SAN myocytes. These records show key differences in the waveforms of the APs of superior and inferior SAN myocytes, including early diastolic duration and early diastolic duration rates. A summary of AP parameters is included in Table 1.

**Table 1.** Summary of AP Parameters in Superior and Inferior SAN Myocytes

	AP Frequency (Hz)	Coefficient of Variation of Inter–AP Interval	Maximum Diastolic Potential (mV)	Takeoff Potential (mV)	Early Diastolic Duration (ms)	Early Diastolic Depolarization Rate (mV/s)	AP Phase 0 Depolarization Rate (mV/ms)	AP Duration at 90% Repolarization (ms)
Superior	4.04 ± 0.36	0.46 ± 0.14	–56.88 ± 1.94	–36.051 ± 1.3	51.36 ± 11.36	83.80 ± 12.86	40.08 ± 8.32	51.56 ± 7.04
Inferior	1.44 ± 0.33	0.96 ± 0.18	–54.63 ± 5.54	–30.99 ± 7.38	325.09 ± 142.38	36.73 ± 7.93	29.67 ± 15.54	75.38 ± 20.65
*t*-test *P-*value	0.0005	0.0375	0.658	0.410	0.031	0.022	0.529	0.219

We found that the frequency of spontaneous APs was significantly higher in the superior versus inferior SAN myocytes (superior: 4.04 ± 0.36 Hz, *n* = 1374 APs from 10 cells; inferior: 1.44 ± 0.33 Hz, *n* = 319 APs from 7 cells; *P* = 0.0028). Although the take off potential of APs (mV) was similar in these cells, the early diastolic depolarization rate was nearly 2.3-fold faster in superior than inferior SAN myocytes. This is reflected in the fact that the early diastolic duration in superior myocytes was only about 0.16 times that in inferior SAN myocytes. These data indicate that, in addition to having higher spontaneous firing frequencies, superior SAN myocytes exhibit a number of intrinsic membrane properties making them more excitable than their inferior counterparts.

In addition to APs, we observed subthreshold voltage fluctuations in superior and inferior SAN myocytes (Figure 3B). The frequency of these subthreshold voltage fluctuations was similar in superior and inferior SAN myocytes (superior: 2.10 ± 0.54 Hz, *n* = 93 subthreshold voltage fluctuations from 5 cells; inferior 1.80 ± 0.33Hz,  *n* = 81 subthreshold voltage fluctuations from 6 cells; *P* = 0.65). However, for superior SAN myocytes, the amplitude histogram of subthreshold fluctuations could be fit with the sum of 2 Gaussian functions (low amplitude events: center at 2.0 mV and width of 1.3 mV; high amplitude center at 11.0 mV and width of 1.1 mV). In contrast, the amplitude histogram of inferior SAN myocytes could be fit with a single Gaussian function (center at 6 mV and width of 2.3 mV). These are consistent with the hypothesis that, at least in superior myocytes, subthreshold voltage fluctuations are produced by different size currents. The differences between superior and inferior SAN myocytes, could be due to a combination of factors, which include regional variations in input resistance and the magnitude of spontaneous currents during diastole in these cells.

We next asked if the occurrence of subthreshold voltage fluctuations followed any notable periodicity or were random by examining the distribution of the timing between these signals. This analysis revealed that the inter-subthreshold voltage fluctuations intervals in both superior and inferior SAN follow a normal distribution and could each be fit with a Gaussian function (superior: center at 333 ms and width of 234.7 ms; inferior: center at 414 ms and width of 217 ms) (Figure 3D). Plotting each inter-subthreshold voltage fluctuations (inter-subthreshold voltage fluctuations_i_) interval against the following one (inter-subthreshold voltage fluctuations_i+1_) (ie, “joint interval plot”)^42^ showed a large degree of dispersion of the data, suggesting independence in the timing between successive subthreshold voltage fluctuations (Figure 3E). These data are consistent with the hypothesis that subthreshold membrane fluctuations occurrence is stochastic.

**Figure 4.**
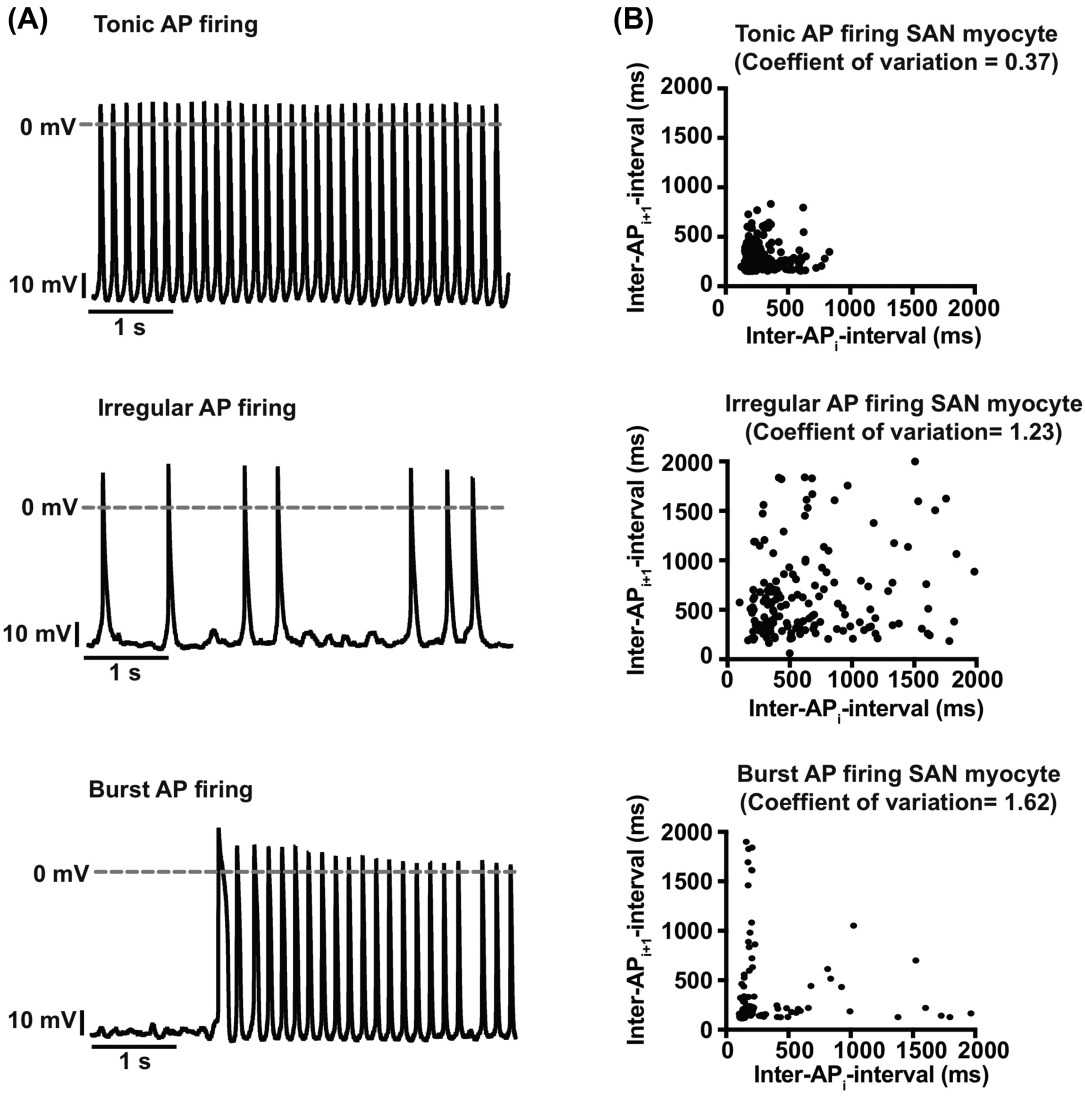
AP firing modalities in superior and inferior SAN myocytes. **(A)** Membrane potential traces from SAN myocytes with tonic (top), irregular (middle), and burst (bottom) AP firing modalities. **(B)** Inter–AP interval plots obtained by plotting each inter–AP interval (inter–AP interval_i_) on the *x*-axis versus the interval of the subsequent AP (inter–AP interval_i+1_) in cells with tonic (top, *n* = 1160 APs from 9 cells), irregular (middle, *n* = 216 APs from 5 cells), and burst (bottom, *n* = 118 APs from 3 cells) AP firing modalities. The coefficient of variation of each AP firing modality is shown at the top of each plot.

A similar analysis was performed for APs to quantify the degree of periodicity of AP firing in superior and inferior SAN myocytes. As part of this analysis, we also determined mean and standard deviation of the inter–AP interval and used them to calculate the coefficient of variation (coefficient of variation = standard deviation/mean) (Figure 4). The rationale for calculating the coefficient of variation was to provide a quantitative measure of inter–AP interval dispersion (ie, periodicity). The higher the coefficient of variation, the lower the periodicity of APs. This analysis revealed that APs in the superior region were more periodic with a significantly smaller coefficient of variation than inferior cells (superior inter–AP interval coefficient of variation = 0.46 ± 0.14, *n* = 1374 APs from 10 cells; inferior inter–AP interval coefficient of variation 0.96 ± 0.18, *n* = 319 APs from 7 cells; *P* = 0.02). It is important to note that a coefficient of variation of 1 (ie, mean = standard deviation) is typically associated with stochastic events. Thus, inferior SAN myocytes seem to fire APs randomly.

To further quantify the diversity in periodic APs between superior and inferior SAN myocytes, we generated joint inter–AP interval plots. SAN myocytes with a regular tonic firing mode have a joint-ISI plot with a tight cluster of intervals, suggesting a high degree of periodicity and dependency in the timing of successive APs (Figure 4A). The mean inter–AP interval was 243.5 ms, standard deviation was 103.7 ms, and coefficient of variation in these cells was 0.37. SAN myocytes with irregular AP firing patterns had joint inter–AP interval plots with a higher degree of data dispersion than in regularly firing tonic cells (Figure 4B). Accordingly, for these cells, the mean, standard deviation, and coefficient of variation of the inter–AP interval were 1003.2 ms, 1222.3 ms, and 1.23, respectively. The combination of high highly dispersed joint inter–AP interval plot and high coefficient of variation value suggest that myocytes with irregular firing patterns are stochastic.

In sharp contrast to regular and irregular AP firing cells, SAN myocytes that generated APs in bursts had an “L-shaped” distribution. This is due to alternating long periods of AP silence with brief periods of higher frequency APs. The mean, standard deviation, and coefficient of variation of the inter–AP interval of “burst” firing cells was 703.6 ms, 1251.4 ms, and 1.62, respectively.

**Figure 5.**
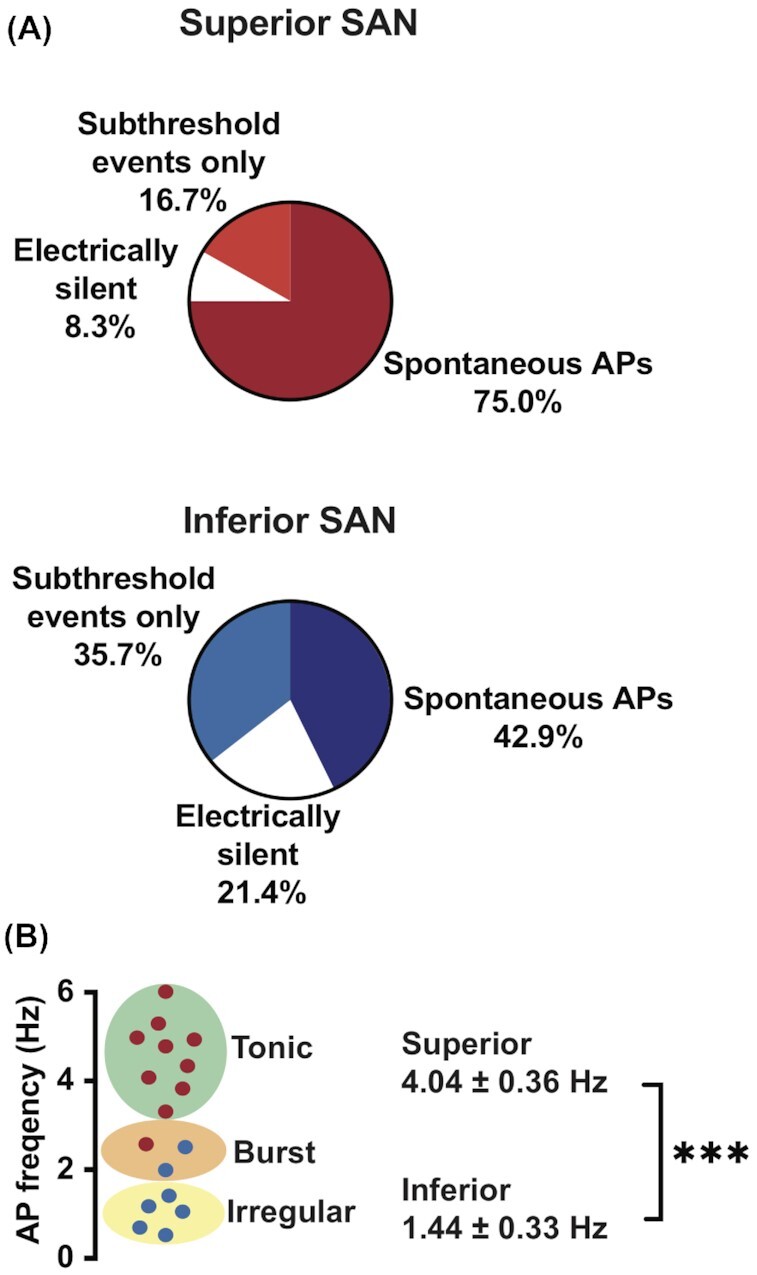
The frequency and periodicity of APs are higher in superior than inferior SAN myocytes. **(A)** Pie charts showing the distribution of electrical signaling modalities in superior (top) and inferior (bottom) SAN myocytes. **(B)** Scatter plot of AP frequencies from superior (*n* = 1374 APs from 10 cells) and inferior (*n* = 319 APs from 7 cells) SAN myocytes, and corresponding distribution among the 2 zones. ***P ≤ 0.001.

Our analysis indicates that the proportion of cells with specific intrinsic-excitability phenotypes varied between the superior and inferior SAN (Figure 5). The pie charts in Figure 5A illustrate that the superior SAN is populated with fewer cells that are electrically silent or produce subthreshold voltage fluctuations and more cells capable of producing APs than inferior SAN. Importantly, most cells in the superior SAN are regularly firing tonic APs while inferior SAN myocytes are mostly irregular, random AP firing modality (Figure 5B).

### SR Ca^2+^ Release Is Necessary for AP Firing but Has No Direct Influence on Subthreshold Voltage Fluctuations in Superior and Inferior SAN Myocytes

**Figure 6.**
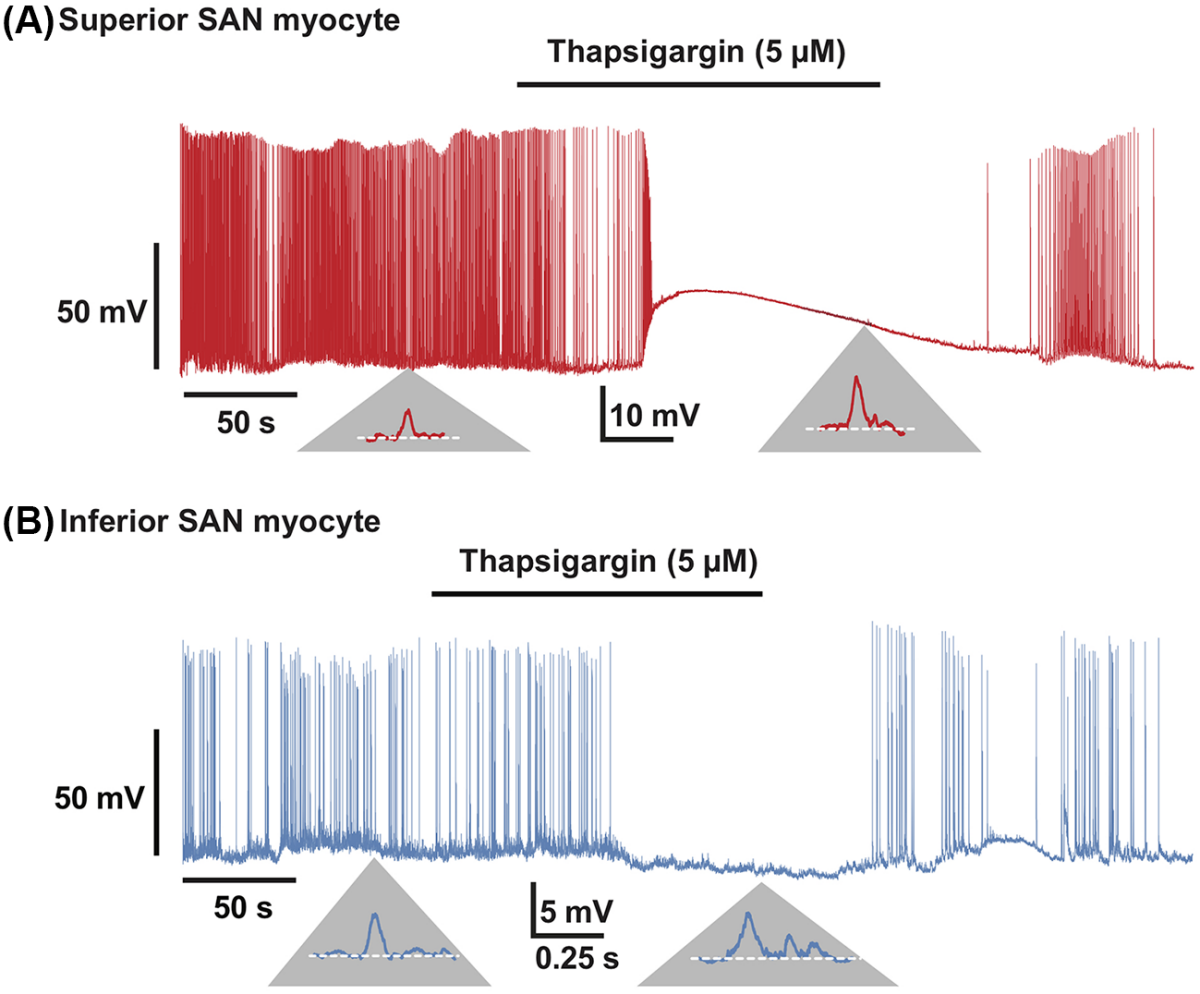
SR Ca^2+^ release is necessary for AP firing but not subthreshold voltage fluctuations in superior and inferior SAN myocytes. Membrane potential traces from representative superior **(A)** and inferior **(B)** SAN myocytes before and after the application of the SR Ca^2+^ ATPase inhibitor thapsigargin (5 μm). Below, traces in panels A and B are zoomed regions of the trace displaying subthreshold voltage fluctuations before and during thapsigargin exposure. Similar results were obtained from 5 superior and 4 inferior SAN myocytes.

We tested the hypothesis that SR Ca^2+^ release contributes to differential electrical activity in superior and inferior SAN myocytes. To do this, we recorded membrane potential before and after the application of the SR Ca^2+^ pump inhibitor thapsigargin (5 μm).^43^ We found that thapsigargin eliminated APs in all superior (*n* = 5 cells) and inferior (*n* = 4 cells) SAN myocytes. Interestingly, inhibiting the SR Ca^2+^ pump differentially altered the resting potential of these cells. Superior SAN myocytes tended to depolarize (observed in 4 out of 5 cells) by + 20 ± 9 mV (Figure 6A), while inferior cells kept their resting potential unaltered or hyperpolarized by a few millivolts (change in membrane potential = –1.5 ± 1 mV) (Figure 6B).

Although thapsigargin stopped AP firing in superior and inferior SAN myocytes, it did not eliminate subthreshold voltage fluctuations. Indeed, the frequency of subthreshold voltage fluctuations was similar in the absence (superior myocytes: 2.10 ± 0.54 Hz; inferior myocytes: 1.80 ± 0.33 Hz) and presence (superior myocytes: 1.98 ± 0.62 Hz; inferior myocytes: 1.73 ± 0.28 Hz; *P* = 0.68) of thapsigargin. Likewise, SR Ca^2+^ pump inhibition did not alter the amplitude of subthreshold voltage fluctuations in superior or inferior SAN myocytes. Superior SAN myocytes produced subthreshold voltage fluctuations with mean amplitudes of 1.3 ± 1.4 mV and 10.9 ± 1.2 mV under control conditions and 2.0 ± 0.5 mV (*P* = 0.3) and 11.0 ± 3.1 mV (*P* = 0.92) during thapsigargin exposure. Similarly, the mean of subthreshold voltage fluctuations in inferior SAN myocytes was unchanged (ie, control = 6.7 ± 1.6 versus thapsigargin = 6.0 ± 2.2 mV, *P* = 0.46) by exposure to thapsigargin.

Removing thapsigargin from the bath restored control AP firing frequency in superior and inferior SAN myocytes, on average, within 107 ± 11 s and 116 ± 14 s, respectively. This is interesting, as thapsigargin is virtually a nonreversible inhibitor of SR Ca^2+^ pump,^43^ suggesting a relatively high turnover rate of SR Ca^2+^ pump protein in these cells. These data suggest that SR Ca^2+^ release is necessary for AP firing, but not subthreshold voltage fluctuations in superior and inferior SAN myocytes.

### Diverse Global and Local Ca^2+^ Signaling Modalities in Superior and Inferior SAN Myocytes

**Figure 7.**
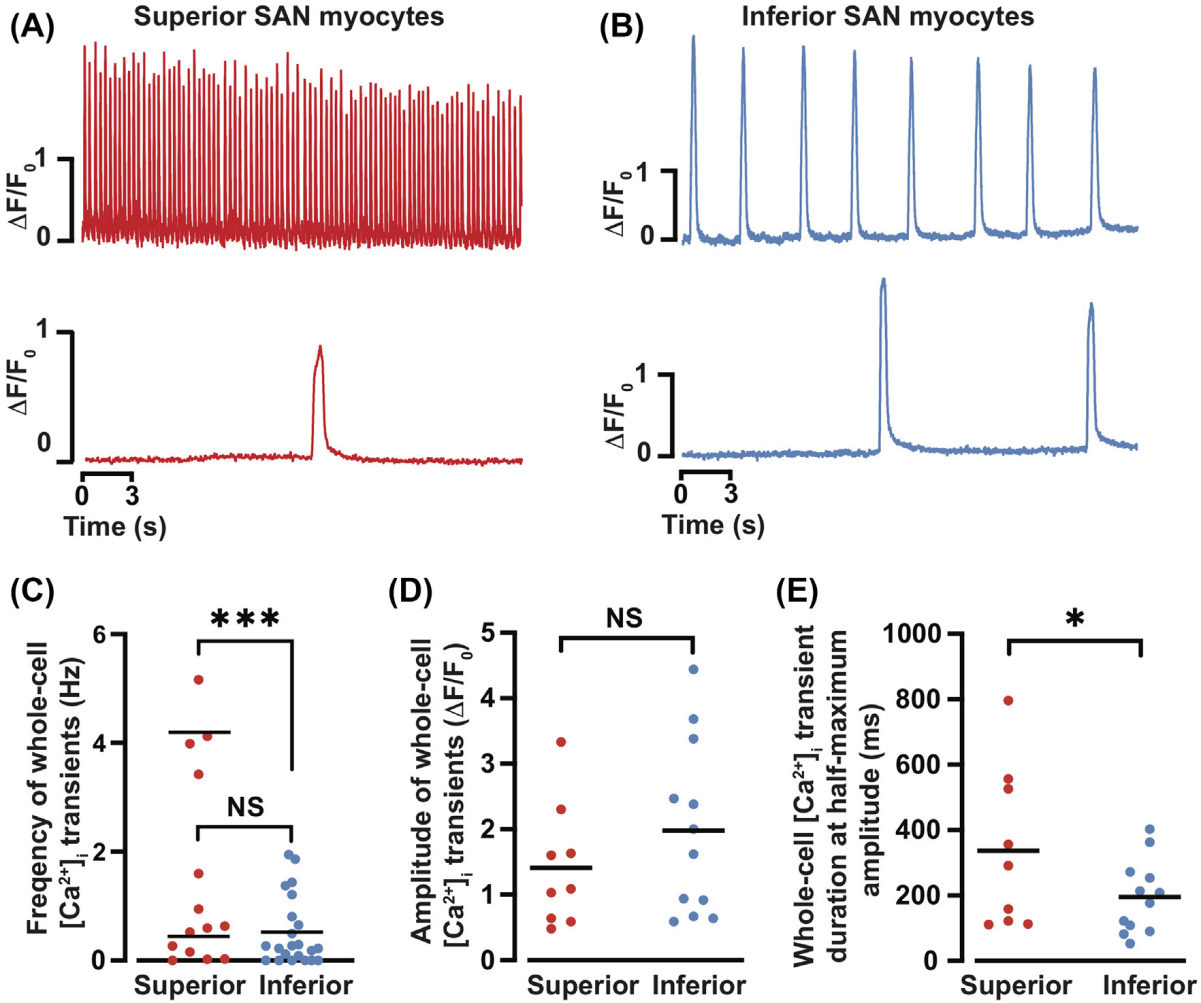
Whole-cell [Ca^2+^]_i_ transients in superior and inferior SAN myocytes. Time course of spontaneous whole-cell [Ca^2+^]_i_ transients from superior **(A)** and inferior **(B)**SAN myocytes. Scatter plots of the [Ca^2+^]_i_ transient frequency **(C)**, amplitude **(D)**, and full duration at half-maximum amplitude **(E)** in superior (*n* = 16 cells) and inferior (*n* = 13 cells) SAN myocytes. NS *P* > 0.05; *P < 0.05; ***P ≤ 0.001.

Next, we visualized superior and inferior SAN myocytes loaded with the fluorescent Ca^2+^ indicator fluo-4-AM (Figure 7). We show the spatially averaged, whole-cell [Ca^2+^]_i_ records from representative superior (Figure 7A) and inferior (Figure 7B) SAN myocytes. Note that, as with APs, we observed a range of Ca^2+^ signaling modalities in these cells: periodic tonic whole-cell [Ca^2+^]_i_ transients, burst, irregular, and cells with local [Ca^2+^]_i_ events only. Notably, while not all SAN myocytes produced whole-cell [Ca^2+^]_i_ transients, all had Ca^2+^ sparks. Indeed, we found that 90% of superior SAN myocytes compared with only 72% of inferior SAN myocytes had whole-cell [Ca^2+^]_i_ transients.

Analysis of whole-cell [Ca^2+^]_i_ transients revealed that the frequency of these events ranged from 0 to 5.16 Hz (*n* = 16 cells) and 0 to 1.95 Hz (*n *= 13 cells) in superior and inferior SAN myocytes, respectively (Figure 7C). The distribution of frequencies of cell-wide [Ca^2+^]_i_ transients in superior SAN myocytes was bimodal. One group of cells fired cell-wide [Ca^2+^]_i_ transients at a frequency of 0.47 ± 0.17 Hz and the other 4.17 ± 0.36 Hz. The frequency of cell-wide [Ca^2+^]_i_ transients inferior SAN myocytes was unimodal and had a mean frequency of 0.52 ± 0.13 Hz, which is statistically similar to that of superior SAN myocytes with low frequency Ca^2+^]_i_ transients (*P* = 0.82). Unlike superior SAN myocytes, inferior SAN myocyte did not produce cell-wide [Ca^2+^]_i_ transients at a frequency higher than 2 Hz.

The amplitude of whole-cell [Ca^2+^]_i_ transients was similar in superior (median ∆F/F_0_ = 1.23; mean ∆F/F_0_ = 1.37 ± 0.18) and inferior (median ∆F/F_0_ = 1.62; mean ∆F/F_0_ = 1.89 ± 0.36; *P* = 0.11) SAN myocytes (Figure 7D).

To determine the rate of clearance of intracellular Ca^2+^, we determined the time to 50% of the [Ca^2+^]_i_ transient (Figure 7E). We found that the full duration at half-maximum amplitude of the global [Ca^2+^]_i_ transient of superior SAN cells (median = 291 ms; mean = 337 ± 81 ms) was longer than that of inferior SAN myocytes (median = 193 ms; mean = 196 ± 32 ms; *P* = 0.04).

**Figure 8.**
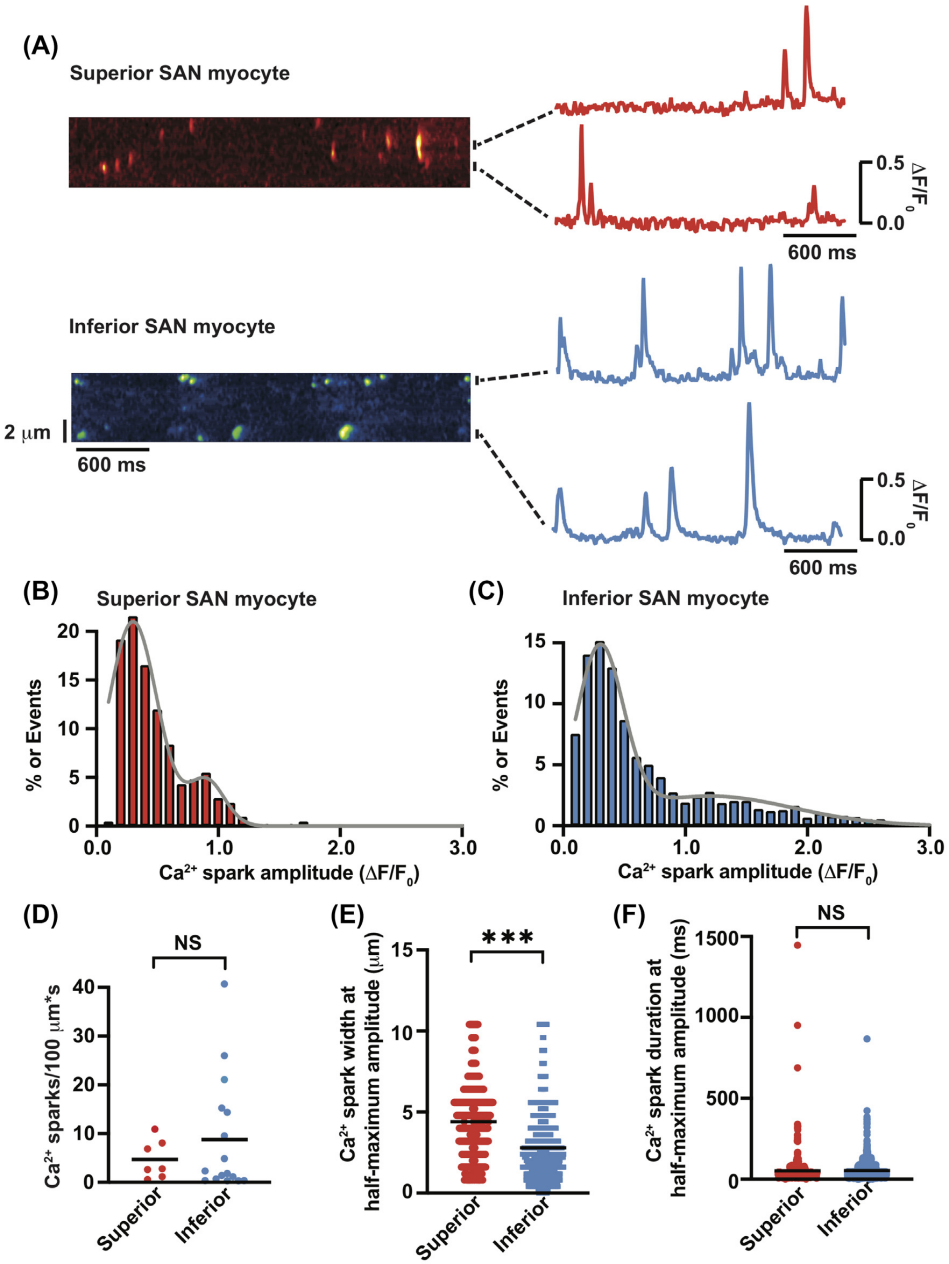
Ca^2+^ sparks in superior and inferior SAN myocytes. **(A)** Exemplar line-scan images of Ca^2+^ sparks in superior (top) and inferior (bottom) SAN myocytes. The traces to the right of each image show the time course of [Ca^2+^]_i_ in the regions identified by horizontal bars. Ca^2+^ spark amplitude histograms in superior (*n* = 2415 sparks from 7 cells)**(B)** and inferior (*n* = 3517 sparks from 16 cells) **(C)** myocytes. Histograms were fit (solid line) with the sum of 2 Gaussian functions. Plots of the Ca^2+^ spark rate**(D)**, full width at half-maximum amplitude **(E)**, and full duration at half-maximum amplitude **(F)** in superior and inferior SAN myocytes. NS *P* > 0.05 and ***P ≤ 0.001.

Next, we examined diastolic Ca^2+^ sparks before diastolic depolarization in both superior and inferior SAN myocytes. Figure 8A shows line-scan kymographs from representative superior and inferior myocytes as well as the time course of [Ca^2+^]_i_ in 2 regions of interest. The amplitude histograms of these diastolic Ca^2+^ sparks (Figure 8B and C) could be fit with the sum of 2 Gaussian functions in both superior and inferior SAN myocytes. On inspection, we found the subset of Ca^2+^ sparks with low amplitudes were similar between superior (∆F/F_0_ = 0.30 ± 0.01; *n* = 2415 events from 7 cells) and inferior (∆F/F_0_ = 0.30 ± 0.02; *n* = 3517 events from 16 cells) SAN myocytes (*P* = 0.57). Notably, the higher amplitude Ca^2+^ sparks are significantly larger in inferior (1.2 ± 0.02) than in superior (0.9 ± 0.01) SAN myocytes (*P* < 0.0001). This analysis is consistent with the view that there are 2 types of Ca^2+^ sparks in superior and inferior SAN myocytes.

Ca^2+^ spark rate ranged from 0.6 to 10.9 events/100 µm*s and 0.3 to 40.7 events/100 µm*s in superior and inferior SAN myocytes, respectively (Figure 8D). The median Ca^2+^ spark rate was 2.8 (mean = 4.7 ± 1.5; *n* = 7 cells) in superior and 2.1 events/100 µm*s (mean = 8.8 ± 2.9; *n* = 16 cells) in inferior SAN myocytes (*P* = 0.19). These Ca^2+^ spark amplitude data indicate that SR Ca^2+^ release sites vary in activity and amplitude between regions of the node, likely reflecting differences in ryanodine receptor cluster sizes and/or SR Ca^2+^ load (ie, driving force).

The spatial spread (ie, full width at half-maximum amplitude; Figure 8E) and duration (ie, full duration at half-maximum amplitude; Figure 8F) was similar in superior and inferior SAN myocytes (*P* = 0.67). Together, this analysis suggests that the geometries as well as clearance mechanisms of Ca^2+^ spark are similar in superior and inferior SAN myocytes.

### Coupling of Global and Local Ca^2+^ Signals to Changes in Membrane Potential in Superior and Inferior SAN Myocytes

A comparison of our electrophysiological and Ca^2+^ data suggests that there is a partial alignment of these signals. For example, inferior SAN myocytes have a single population of subthreshold voltage fluctuations (Figure 3C), but a bimodal Ca^2+^ spark amplitude distribution (Figure 8C). Furthermore, although superior and inferior SAN myocytes have a subgroup of Ca^2+^ spark with similar amplitudes, their subthreshold voltage fluctuations are significantly different. Similarly, while there were more superior cells with higher frequencies of whole-cell [Ca^2+^]_i_ transients than inferior cells, we did not detect the same level of segregation or similarity in frequencies in APs (Figure 5B). This could be due to some single [Ca^2+^]_i_ transients caused by a train of APs. Together, these data suggest a lack of 1:1 correspondence of APs and whole-cell [Ca^2+^]_i_ transients as well as potential variations in coupling strength between Ca^2+^ sparks and changes in membrane potential between superior and inferior SAN myocytes.

**Figure 9.**
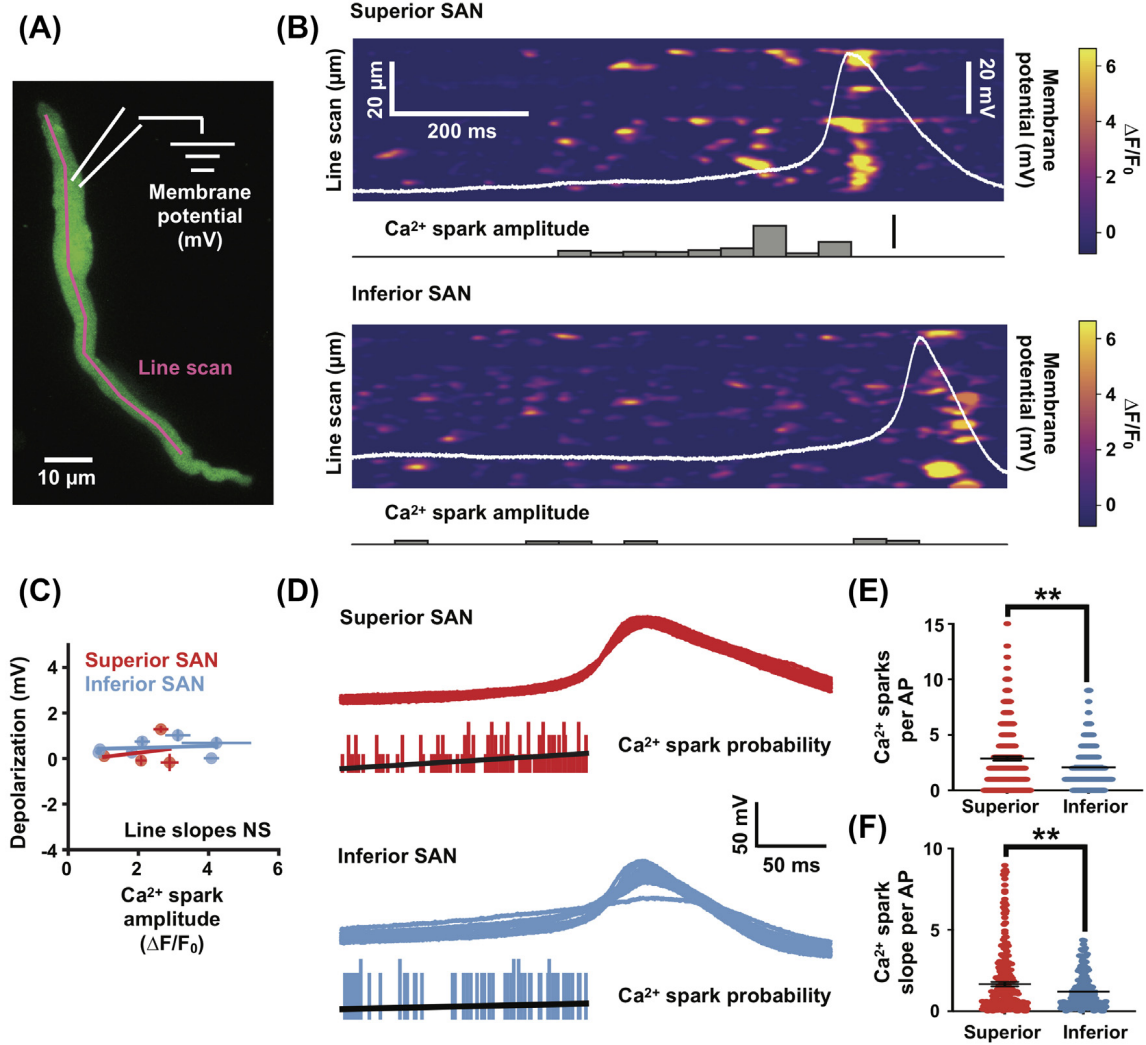
Weak coupling between individual Ca^2+^ sparks to membrane potential in superior and inferior SAN myocytes. **(A)** Schematic showing experimental configuration to perform simultaneous whole-cell recording of membrane potential (mV) and [Ca^2+^]_i_ imaging (line scan) in isolated SAN myocytes. The image is a maximal intensity projection of a time series (30 s) of a SAN myocyte loaded with the Ca^2+^ indicator Fluo-4 (green). [Ca^2+^]_i_ activity is monitored with a repeated line scan (magenta) while simultaneously recording membrane potential with a whole-cell pipette (membrane potential). **(B)** Representative dual recordings of a superior (upper) and inferior (lower) SAN myocytes. Each image is a kymograph of line scans (vertical axis) repeated every ∼4 ms for a total of 10 000 scans (horizontal axis). The intensity values in the image are expressed in ∆*F*/*F*_0_. Simultaneous whole-cell recording of membrane potential (mV) is overlaid (in white). The Ca^2+^ spark amplitude histogram (lower) is constructed by extracting Ca^2+^ sparks from the kymograph and represents their binned amplitude. **(C)** Scatter plot comparing the amplitude of Ca^2+^ spark with the resultant membrane depolarization in both superior (red) and inferior (blue) dual-recorded SAN myocytes. Each point is the mean ± standard error of the mean for individually recorded cells. **(D)** Multiple whole-cell APs aligned to their time of threshold (black traces, *n* = 15 APs) from both superior (upper) and inferior (lower) dual-recorded SAN myocytes. Histogram (gray) is the probability of Ca^2+^ sparks before an AP. Line (blue) is the linear fit of the probability of Ca^2+^ sparks leading to an AP. **(E)** Plot of the number of Ca^2+^ sparks per AP from superior (*n* = 4 cells) and inferior (*n* = 6 cells) SAN myocytes. **(F)** Plot of the slope of Ca^2+^ sparks before each AP from superior and inferior SAN myocytes. **P ≤ 0.01.

To address this issue, we simultaneously recorded membrane potential and [Ca^2+^]_i_ from superior and inferior SAN myocytes to examine the relationship between the occurrence of Ca^2+^ sparks and the resultant impact on membrane potential depolarization. We performed dual recordings from SAN myocytes by simultaneously monitoring [Ca^2+^]_i_ with confocal line scans of cells loaded with Fluo-4 paired with whole-cell current clamp recording (Figure 9A). Visually inspecting these dual recordings of Ca^2+^ sparks and whole-cell membrane potential we did not see pronounced effects on membrane potential before, during or after individual Ca^2+^ sparks (Figure 9B). To test if the occurrence of Ca^2+^ sparks had an impact on membrane potential depolarization, we quantified the relationship between Ca^2+^ spark amplitude and the resultant membrane potential depolarization and found no significant correlation (Figure 9C; superior *r*^2^ = –0.068, *n* = 4 cells, *P* = 0.93; inferior *r*^2^ = 0.453, *n* = 6 cells, *P* = 0.37).

If individual Ca^2+^ spark produce little to no membrane potential changes between APs, is the pattern of Ca^2+^ sparks leading to an AP different between the superior and inferior regions? To examine this, we asked if the number of Ca^2+^ sparks or the slope of Ca^2+^ sparks approaching an AP was different between superior and inferior SAN cells (Figure 9D). Interestingly, we found significantly more Ca^2+^ sparks leading to an AP in the superior versus the inferior SAN (Figure 9E; superior 4.5 ± 2.8 Ca^2+^ sparks, inferior 3.4 ± 1.7 Ca^2+^ sparks, *P* = 0.006). Likewise, we found a significantly steeper slope in Ca^2+^ spark accumulation before each AP in superior versus inferior SAN myocytes (Figure 9F; superior 2.0 ± 2.1 Ca^2+^ spark slope; inferior 1.2 ± 1.1 Ca^2+^ spark slope, *P* = 0.007).

This analysis indicates that while the coupling of individual Ca^2+^ sparks to membrane potential depolarization is weak throughout the SAN, there is a regional difference in the number and rate of accumulation of Ca^2+^ sparks before an AP. With the superior region receiving a stronger driving force of Ca^2+^ sparks leading to AP generation. This could be a mechanism contributing to the higher spontaneous AP frequency of superior versus inferior myocytes.

## Discussion

We generated high-resolution 3D maps of the vasculature and SAN myocytes across the entirety of the mouse SAN. Our data show that vascular and myocyte densities vary regionally along the SAN. The superior SAN is highly vascularized, creating a redundant network whereby all pacemaking myocytes are near at least 1 vessel. This affords a high surface area for exchange and minimizes the diffusional distances in a region of the node where myocytes have highest intrinsic firing rates. In contrast, vascular and myocyte densities are lowest in the inferior SAN. The inferior SAN is also where myocytes with the lowest inherent AP firing rate are located. Thus, the SAN seems to be designed so that vascular density varies regionally to match the electrical signaling modalities of the myocytes that populate this heterogenous tissue. An important implication of these findings is that regional variations in blood supply could be a key factor determining the site of AP origin in the SAN as well as its firing frequency.

Based on our detailed vascular and myocyte anatomical and functional data, we propose a new, objective identification of the superior and inferior mouse SAN. Our data indicate that SAN myocyte-to-vessel distance is relatively short and remains relatively similar from the initiation point of the SAN artery until about 3.25 mm toward the inferior section of the node. We call this region the superior SAN. We propose that the starting point of the inferior SAN is set at the lowest point of the superior node, where there is a relatively sharp, nearly 1.5-fold increase in myocyte-to-vessel distance. Using this framework, the superior and inferior SAN account for about 60% and 40% of the mouse node, respectively.

An interesting observation in our study was that while the diameter of the SAN artery decreased nearly 20% from the superior to inferior SAN, the diameter of 2°–4° vessels did not. Thus, differences in vascular density are not due to fundamental differences in the structure of microvessels, rather their number.

Our work is consistent with a recent study from the Efimov lab^23^ involving rat hearts. They found that, although the superior and inferior sections of the node have the capacity to fire action potentials, the superior node has the fastest firing rate and hence functions as the dominant pacemaking center in of the SAN. The rat inferior SAN was capable of becoming the dominant pacemaking center, but only when the superior node was electrically silenced. Although the concept of a functional heterogeneous SAN has been proposed before, our study, in combination with Brennan et al.,^23^ supports a model where heterogeneity encompasses all elements of nodal function, ie, vascular, neuronal, and myocyte, but in 2 distinct regions of the node, at least in rodent hearts.

What is the physiological significance of the reciprocal association of high excitability with high vascular density in the SAN? Our study, in combination with others, provide a potential answer to this difficult question. A reasonable hypothesis is that vessel density and hence blood supply scales up with tissue metabolic needs. It is estimated that a neuron consumes about 7–9 million ATP molecules during a single AP.^44,45^ At low firing rates (ie, 0.1 Hz), ATP levels in neurons remain relatively stable, suggesting a balance between ATP synthesis and hydrolysis. However, increasing AP frequency (>10 Hz) leads to rapid ATP depletion within seconds of the onset of high-frequency stimulation as ATP consumption exceeds production.^46^

Our electrophysiological data suggest that the intrinsic AP frequency is about 3.9-fold higher in superior versus inferior SAN myocytes. Although similar ATP consumption studies like those in neurons have not been performed in SAN myocytes, assuming that energetic cost of firing an AP is likely similar in these cells, our data suggest that in terms of APs alone, ATP consumption could be 3.9-fold higher in superior compared with inferior SAN myocytes. Note, however, that a relatively large fraction the inferior SAN myocytes do not produce APs. Thus, the energetic differential between superior and inferior SAN myocytes could be even larger than 3.9-fold in these cells.

Although neurons and SAN myocytes have the capacity to spontaneously fire APs, their energetic needs are likely quite different. As in neurons, these include ion transport across the sarcolemma and intracellular organelles such as the endoplasmic reticulum.^24^ Additional ATP is consumed by myosin-actin crossbridge cycling and the constant generation of cytosolic cAMP by adenylate cyclase.^25^ The latter is significant as our data show that even the fastest firing superior SAN myocytes produce APs at a lower rate than the heart rate of a mouse, which is about 12–13 Hz.^47,48^ This disparity between in vivo and isolated single cell firing rates reflects strong input from the sympathetic arm of the autonomic nervous system, which increases firing rate through the activation of cAMP/protein kinase A signaling. Indeed, recent studies by Brennan et al.^23^ and Hanna et al.^49^ suggested that sympathetic innervation and drive is larger in the superior compared with the inferior SAN. Thus, a combination of higher AP firing, Ca^2+^ cycling, and contraction rates as well as cAMP production may conspire to produce a much larger energetic load in superior than inferior SAN myocytes. In combination with our vascular mapping, this implies that—as originally proposed Krogh^50^ for skeletal muscle—microvessel density scales up with the energetic needs of SAN myocytes.

Although performing a detailed analysis of the electrophysiological properties of superior and inferior SAN myocytes was beyond the scope of this study, our data provide insights into this interesting issue. We found that the coupling strength between Ca^2+^ sparks and membrane potential is relatively weak, but similar in superior and inferior SAN myocytes. However, the number of Ca^2+^ sparks immediately before an AP was larger in superior than inferior SAN myocytes. Thus, increased SR Ca^2+^ release could contribute to faster diastolic depolarization rates leading to AP threshold in superior myocytes and hence higher firing rates. In principle, these regional variations in Ca^2+^ clock function could be the result of differences in the activity of Ca^2+^ channels, SR Ca^2+^ pump, ryanodine receptors, and/or Na^+^/Ca^2+^ exchanger. Future experiments should determine whether regional differences in the function and expression of these proteins as well as HCN^51^ and Ca^2+^ currents contribute to differential electrical firing patterns across the SAN.

An interesting finding in this study is that SR Ca^2+^ pump inhibition with thapsigargin eliminated AP firing, but not subthreshold voltage fluctuations in superior and inferior SAN myocytes. In combination with our finding that Ca^2+^ sparks are weakly coupled to changes in membrane potential, this suggests that SR Ca^2+^ release may be necessary, but not sufficient to trigger an AP in superior and inferior SAN myocytes. Furthermore, our data suggest that subthreshold voltage fluctuations are unlikely produced by Ca^2+^ spark activation of Na^+^/Ca^2+^ exchanger currents. In a follow-up study, we will investigate the molecular identity of the proteins underlying subthreshold voltage fluctuations.

Finally, we formulate a new model for pacemaking activity that integrates our functional and anatomical mapping with the stochastic resonance model of SAN firing proposed by Clancy and Santana^52^ and Bychkov et al.^15^ The model is illustrated in the graphical abstract of this paper. In this model, all SAN myocytes function like a bistable system, alternating from the maximum diastolic potential to a full AP. SAN myocyte in the sparsely vascularized inferior section of the node fires stochastic subthreshold voltage fluctuations and sporadic APs. These events do not lead to periodic pacemaking on their own. However, when coupled to a more periodic voltage oscillators such as superior SAN myocytes, subthreshold voltage fluctuations and rare APs integrate (ie, resonance) and increase the probability that superior SAN myocytes reach AP threshold. Thus, inferior SAN myocytes likely increase the strength and periodicity of superior SAN activation and hence pacemaking activity. Stochastic resonance models have also been implicated in the periodic activation of APs in neurons.^53,54^ A key new insight provided by our data is that the capacity of specific regions of the node to operate as periodic oscillators or stochastic signal generators could be critically dependent on vessel density and hence blood flow. Thus, blood supply could effectively determine the dominant pacemaking site within the SAN.

## Data Availability

The data underlying this article will be shared on reasonable request to the corresponding author.
